# AI Privacy and Security in Healthcare: A Systematic Literature Review

**DOI:** 10.63144/ijt.2065.6747

**Published:** 2026-06-01

**Authors:** Diane Dolezel, Karima Lalani, Valerie Watzlaf, Kerryn Butler-Henderson, Elise V.Z. Lambert, Mary Morton, Jamie Sand, David Gibbs, Susan Fenton

**Affiliations:** 1 Health Informatics & Information Management Department, Texas State University, Round Rock, Texas, USA; 2 Health Informatics and Health Information Management Program, School of Public Health, University of Washington, Seattle, Washington, USA; 3 Department of Health Information Management, School of Health and Rehabilitation Sciences, University of Pittsburgh, Pittsburgh, Pennsylvania, USA; 4 Charles Sturt University, Wagga Wagga, New South Wales, Australia; 5 University of Mississippi Medical Center, Health Informatics and Information Management, Jackson, Mississippi, USA; 6 School of Public and Population Health, Boise State University, Boise, Idaho, USA; 7 McWilliams School of Biomedical Informatics, The University of Texas Health Science Center at Houston, Houston, Texas, USA

**Keywords:** Artificial intelligence, Privacy, Security, Systematic review, Telerehabilitation

## Abstract

**Background:**

Artificial intelligence is expanding into telemedicine and telerehabilitation, yet significant privacy and security concerns persist.

**Scope:**

To synthesize empirical evidence on privacy and security approaches in health care, particularly those relevant to distributed home care.

**Methodology:**

A systematic review identified 80 studies (2019 to 2025), and Latent Dirichlet Allocation (LDA) topic modeling characterized the privacy and security themes.

**Results:**

Sixty-six studies addressed privacy, only seventeen addressed security, and three studies addressed both. LDA identified four themes: patient data privacy, federated learning for medical imaging, encrypted training and secure computation, and healthcare data governance. Most studies emphasized privacy-preserving approaches, like federated learning, encryption, and differential privacy. Almost half were conducted outside healthcare environments, limiting insight into real teleclinical and telerehabilitation workflow.

**Conclusion:**

Securing healthcare AI will require a multi-layered governance framework, broader global representation, and integration of privacy and security protections into routine clinical workflows.

Artificial intelligence (AI) is increasingly integrated into clinical workflows, from prediction and image analysis to clinical decision support and remote care, which is expanding access but creating new exposure points for privacy and security. Privacy refers to a person’s ability to determine when, how, and for what purposes their health information is collected, shared, and used (U.S. Department of Health & Human Services, 2023a). In healthcare, security is operationalized through administrative, technical, and physical safeguards that protect information systems and preserve confidentiality, integrity, and availability (U.S. Department of Health & Human Services, 2023b). Because AI systems rely on large, distributed, and continuously generated health data, these distinctions are important for governance and oversight. Despite a growing focus on AI innovation, privacy and security challenges specific to AI-enabled healthcare, especially in remote care contexts, remain fragmented and insufficiently explored.

## AI Applications and Remote Health Care

In telehealth and telerehabilitation settings, AI extends care delivery beyond traditional clinical environments by supporting remote monitoring, clinical decision-making, documentation, and personalized care outside the hospital (Li, Li, Wei, & Li, 2024). AI applications include virtual assistants, wearable-based monitoring systems, predictive models of disease progression, personalized treatment recommendations, and AI-enabled clinical scribes (Chandrasekaran & Moustakas, 2025). As sensitive data flows through home networks, personal devices, and third-party platforms, privacy and security risks increase (Houser et al., 2023). Remote sensors and cloud-based systems further expand the attack surface for system-level vulnerabilities and coordination failures, highlighting the need for robust security and governance measures (Singh et al., 2025).

## Privacy, Security, and Patient Trust in Remote Care

Successful adoption of AI-enabled telehealth depends on patient trust, particularly transparency in data collection and use (Chan et al., 2025; Chew & Achananuparp, 2022; L. Li et al., 2024). Although patients acknowledge AI’s potential benefits, privacy concerns and limited understanding often reduce their willingness to share health data (Aggarwal et al., 2021; Davis et al., 2025; Khullar et al., 2022). For instance, while over half of U.S. respondents believed AI could improve care, most expressed privacy concerns, with similar patterns in Canada and the United Kingdom, where fears of re-identification further limited data sharing (Aggarwal et al., 2021; Davis et al., 2025; Khullar et al., 2022). Acceptance varies by application, with greater hesitation toward AI documentation tools but higher perceived privacy when using chatbots for sensitive topics (Chandrasekaran & Moustakas, 2025; Dellavelle et al., 2025). Prior reviews highlight gaps in usability, accountability, informed consent, and equity, underscoring the need for patient-centered co-design in AI governance (Moy et al., 2024).

## Governance and Regulatory Challenges for Healthcare AI

In this review, governance refers to the policies, accountability structures, and operational controls that translate ethical principles into enforceable privacy and security practices. Despite regulatory efforts driven by patient concerns and organizational risk, the effectiveness of AI-enabled telehealth in addressing privacy and security challenges remains unclear. Chan et al. (2025) identified over 200 AI ethics frameworks but found limited real-world impact, particularly in translating principles into enforceable governance, security, and accountability mechanisms, leaving gaps in oversight for data misuse and AI-related harm.

Telehealth and telerehabilitation increases risk by extending data flows beyond clinical settings to personal devices, home networks, and third-party platforms. Regulatory oversight varies internationally: the American Telemedicine Association provides telehealth-specific guidance (American Telemedicine Association, 2025), whereas Software as a Medical Device is governed by region-specific frameworks, including the EU Medical Device Regulation, Australia’s Therapeutic Goods Act, U.S. Food and Drug Administration guidance, and South Korea’s Digital Medical Products Act (Dobson & Ruhl, 2025). International bodies such as the World Health Organization and the National Academy of Medicine have also proposed ethical frameworks that promote fairness and accountability (Aggarwal et al., 2021; Kelly et al., 2019; Tsoi, 2025).

## Gaps in Literature and Rationale for Review

Most prior research has focused on AI adoption, public perceptions, and high-level governance frameworks, with comparatively less emphasis on privacy and security risks in AI-enabled healthcare. Studies highlight persistent mistrust, including concerns about unauthorized data collection from voice-activated devices (Chew & Achananuparp, 2022), and show that fewer than 25% of AI implementations report using privacy or security controls (Aravazhi et al., 2025). Although some reviews examine clinician cybersecurity duties or technical safeguards (Elendu et al., 2024; Shojaei et al., 2024), they do not address risks specific to AI-driven systems. These gaps point to the need for methods that can synthesize diverse evidence and systematically identify recurring privacy and security issues. Combining Latent Dirichlet Allocation (LDA) with PRISMA provides a scalable, data-driven approach to detecting key themes and trends in this literature.

### Research Questions

Guided by this combined systematic and data-driven approach, this review addresses the following research questions:

1. What are the key privacy and security themes in healthcare AI literature?
2. How can LDA enhance the identification of privacy and security themes in healthcare AI literature?

These findings are intended to inform policymakers, health informatics professionals, and AI developers seeking to deploy AI responsibly in telehealth and telerehabilitation settings.

## Methods

The review process was organized in alignment with PRISMA guidance to ensure clear, comprehensive, and reproducible reporting (Page et al., 2021).

### Eligibility Criteria

To be included, studies had to present original empirical findings, be published in English between 2018 and 2025, and focus on privacy or security issues within healthcare-related AI. Publications that did not provide primary data, such as systematic reviews, meta-analyses, dissertations, theses, or editorial and opinion pieces, were excluded to ensure the review synthesized only firsthand evidence. Non-English studies were also omitted to support consistency in coding and interpretation.

### Information Sources and Search Strategy

A comprehensive search strategy was developed to identify relevant studies across seven databases: CINAHL, EBSCO, Ovid, PubMed, ScienceDirect, Scopus, and Web of Science. The year 2018 was selected as the start date, as it marks a period in which the adoption of AI in healthcare began to accelerate substantially (Jackson & Hu, 2019). Search terms were designed to capture literature related to healthcare AI privacy and security issues, using this Boolean string:

[artificial intelligence OR automation OR augmented] AND
[privacy OR security] AND
[health OR clinical OR clinician OR medical OR medicine OR nurse OR nursing OR patient].

### Selection Process

Two members of the nine-person review team (DD, KL, EL, VW, JS, KBH, MM, DG, JM) independently screened each title and abstract using the predetermined inclusion and exclusion criteria, resolving differing assessments through discussion. Full-text evaluation was then conducted by reviewer pairs drawn from a group of seven reviewers (DD, KL, JS, MM, VW, EL, DG), with any unresolved differences referred to a third reviewer for final judgment. Study details, such as privacy or security focus, target population, intervention characteristics, outcomes, and major findings, were recorded using a standardized extraction template within Covidence® (Veritas Health Innovation, n.d.).

### Data Extraction

All identified records were imported into Covidence®, an online platform for systematic review management, where duplicates were removed. The data extraction form was pilot tested by two reviewers before full extraction to ensure consistency across reviewers.

### Thematic Analysis

Eighty studies met the eligibility criteria and were exported as Word files for processing in R, where text documents were prepared using the *topicmodels* package. LDA was then applied to uncover dominant themes by examining patterns of word co-occurrence across the text body (Blei et al., 2003; Feller et al., 2018). Preprocessing steps involved routine text-cleaning procedures, including the removal of punctuation and stop words. Multiple model configurations were tested, and a four-topic solution was selected because it offered the most coherent and interpretable structure. Two reviewers independently reviewed the LDA outputs and refined the topic labels, organizing them into broader thematic categories based on salient terms and conceptual alignment. The use of LDA in this context is consistent with prior healthcare research that employs topic modeling to analyze clinical narratives and other unstructured text sources (Danler et al., 2024; Sun et al., 2024). A formal risk-of-bias assessment was not performed, given the substantial heterogeneity in study designs and the exploratory aims of the analysis.

## Results

### Study Selection

**Figure 1:**
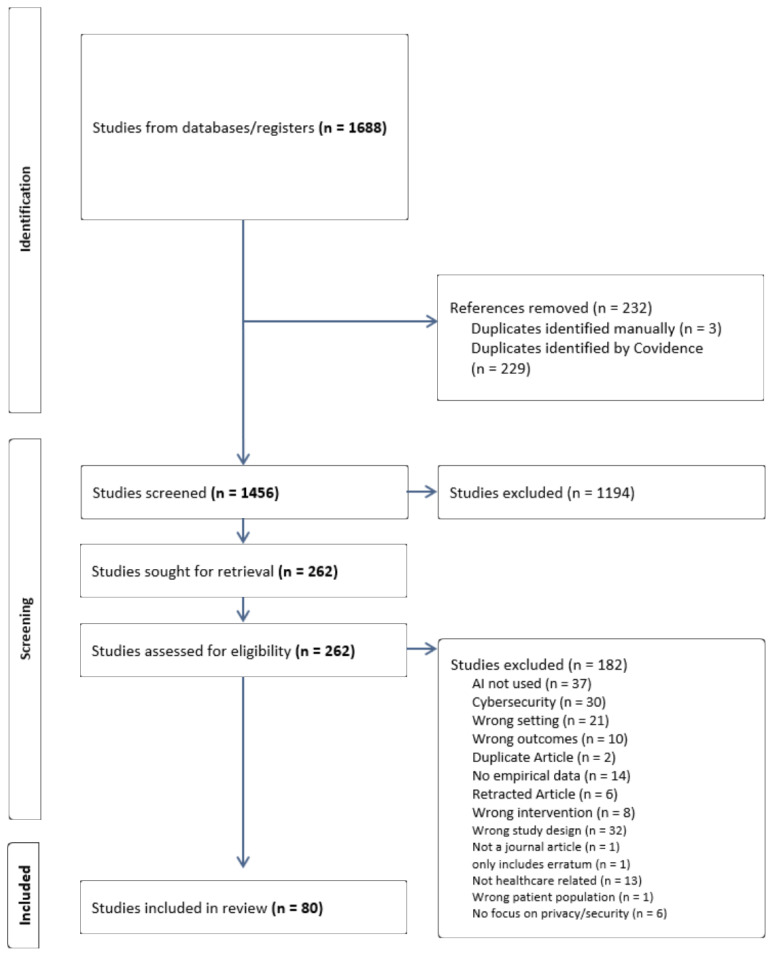
PRISMA Study Selection Workflow

Figure 1 summarizes the PRISMA study selection workflow. The database search yielded 1,688 records, of which 232 duplicates were removed before screening. The remaining 1,456 titles and abstracts were assessed, of which 1,194 did not meet the inclusion criteria. A total of 262 full-text articles were reviewed, and 182 were excluded for reasons such as absence of an AI component (n = 37), unsuitable study design (n = 32), a focus limited to cybersecurity rather than privacy or security (n = 30), an ineligible study setting (n = 21), or lack of empirical data (n = 14). The other 48 studies were excluded due to many different reasons as can be seen in Figure 1. Ultimately, 80 studies met all criteria and were included in the final analysis.

## Characteristics of Included Studies

Table 1 provides an overview of the study characteristics, including design type, study setting, and publication year for all 80 included studies. The studies varied by design, setting, and publication year.

Study designs were grouped according to the primary methodological approach described by each article. Experimental research was the most common (n = 45, 56.2%), followed by model development (n = 12, 15.0%). Case studies and pilot investigations were less frequent (each n = 4, 5.0%), as were cohort and cross-sectional designs (each n = 3, 3.8%). A small number of studies employed alternative approaches such as algorithmic development, qualitative methods, mixed-methods designs, or exploratory formats, and one study did not specify a design.

Study settings varied widely, with nearly half of the research conducted outside of traditional healthcare environments (n = 37, 46.2%). Hospital studies accounted for 17.5% (n = 14). Thirteen articles (16.2%) did not report on their study setting.

### Distribution of Included Studies by Year

**Figure 2:**
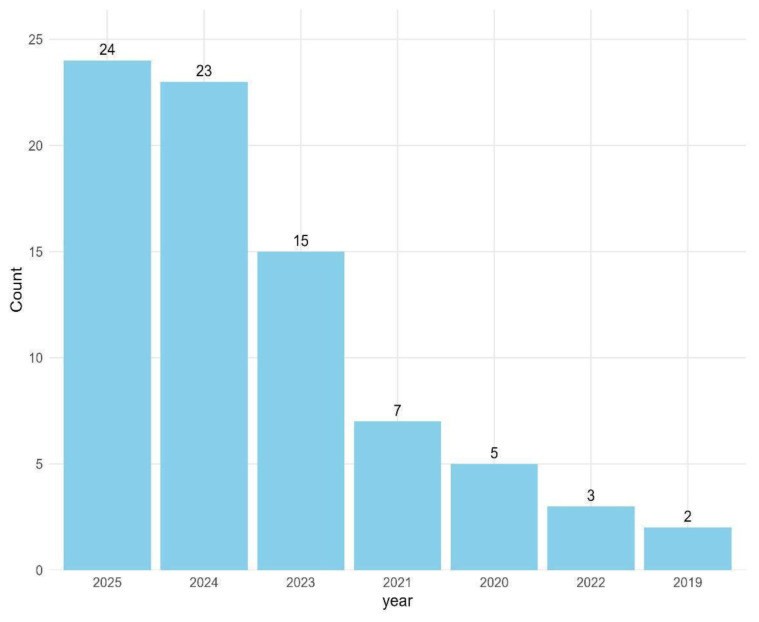
Distribution of Studies by Publication Year, 2019–2025

Figure 2 displays the distribution of studies published by year from 2019 to 2025. Study publications increased over time, with peaks in 2024 (n=23) and 2025 (n=24). Fewer studies were published before 2023, particularly in 2019 (n = 2) and 2022 (n = 3). One study did not report a publication year.

### Descriptive Summaries of Journals Reviewed

The Appendix (Table A1) presents a detailed summary of the 80 studies examining privacy and security content from 2019 to 2025. Although the search strategy included studies from 2018 onward, no studies published in 2018 met the inclusion criteria. Among the 80 included studies, 66 (82.5%) addressed privacy and 17 (21.25%) explored security, with three of these studies addressing both topics.

### Geographic Distribution of Included Studies

Table 2 summarizes the geographic distribution of the included studies. The table reports the number of studies conducted in each country, noting that some projects involved multiple international sites; therefore, frequencies are presented as counts rather than percentages. Research activity was concentrated primarily in Asia and the Middle East. China contributed the largest number of studies (n = 25), followed by India (n = 16) and Saudi Arabia (n = 10). The United States accounted for six studies, while Canada and Germany each contributed five, and South Korea contributed four. Several additional countries, including Bangladesh, Finland, Jordan, Malaysia, the Netherlands, Pakistan, and Taiwan, were represented by two studies each. The remaining countries, Algeria, Australia, Austria, Belgium, Hong Kong, Oman, Portugal, Romania, Slovakia, South Africa, Turkey, the United Kingdom, and Yemen, each appeared in one study.

## LDA Findings

After summarizing study characteristics and publication patterns, we applied LDA to uncover underlying themes in the literature. The LDA topic modeling identified four themes, each characterized by its most frequent keywords:

1. Patient Data Privacy in ML Models (keywords: data, privacy, learning, model, health, dataset).
2. Federated Learning for Medical Imaging Privacy and Performance (keywords: federated, privacy, training, performance, medical, image).
3. Encrypted Medical Imaging and Secure Training (keywords: encryption, privacy, learning, training); and
4. Healthcare Data Privacy and System Governance (keywords: healthcare, system, security, information).

Federated learning is discussed in multiple thematic areas, including decentralized training, privacy protection, and secure collaboration frameworks.

## Thematic Results

### Theme 1: Patient Data Privacy in ML Models

The first theme centers on the protection of patient information within healthcare AI systems, a dominant concern across the included studies. Terms such as *privacy*, *data*, *health*, *information*, and *learning* frequently appeared, reflecting the strong emphasis on safeguarding sensitive clinical details. A range of privacy-preserving techniques were described, including federated learning to support decentralized model development for applications such as disease prediction, mental-health assessment, EHR exchange, and medical-imaging analysis (Choudhury et al., 2025; Dubey et al., 2025; Lee et al., 2024; Om Kumar et al., 2023; Shao et al., 2020; Xu et al., 2022). Additional strategies, such as differential privacy, blockchain infrastructures, homomorphic encryption, k-anonymity, and entropy-based methods, were also used to minimize re-identification risks (Alabdulwahab et al., 2024; Benouis et al., 2024; Hennebelle et al., 2024; Kokin et al., 2024; Sangaiah et al., 2024; S. Wang et al., 2025).

Even with these safeguards, literature highlights several vulnerabilities that remain unresolved. Studies have shown that word-embedding models can inadvertently expose patient details (Abdalla et al., 2020), large language models may reveal protected information under certain conditions (Chuang et al., 2025), and behavioral-biometric data can still enable individual re-identification (Deepak et al., 2024; Vignesh et al., 2025). To mitigate these risks, researchers increasingly advocate for local model deployment or the use of open-source, privacy-preserving tools that limit or avoid external data transfer (Nowak et al., 2025; Seyfizadeh et al., 2024; Y. Zhang et al., 2025).

### Theme 2: Federated Learning for Medical Imaging Privacy and Performance

The second theme emphasizes the prominent role of federated learning (FL) in supporting privacy-preserving development of medical-imaging AI models. The frequent appearance of keywords such as *federated*, *privacy*, *medical*, and *image* reflects how strongly FL is associated with decentralized analytics across healthcare institutions. By enabling algorithms to be trained locally at each site while sharing only model parameters, FL provides a practical solution for multi-institutional collaboration in contexts where direct data sharing is restricted by ethical, legal, or regulatory requirements. This approach has been used across a wide range of imaging applications, including skin cancer classification, tumor characterization, ophthalmic disease detection, polyp segmentation, sleep-stage recognition, ECG analysis, and COVID-19 assessment (Ain et al., 2023; Baumgartner et al., 2023; Butt et al., 2023; Hasan & Rahman, 2025; Li et al., 2025; Yahiaoui et al., 2024; Yan et al., 2023).

A consistent challenge across these studies is the substantial heterogeneity in data distributions between institutions, which can degrade model performance when training is performed locally. Researchers have addressed this issue using strategies such as domain adaptation, transfer learning, and personalized aggregation mechanisms designed to adjust model parameters to site-specific characteristics (Bangare et al., 2024; Gulati et al., 2025; Haripriya et al., 2025; Tang et al., 2024). Many FL implementations also introduce additional privacy safeguards, including differential privacy, synthetic data augmentation, and noise-based perturbation, to reduce the risk of re-identification and to support learning from scarce or imbalanced datasets (Hasan & Rahman, 2025; He et al., 2024; Iqbal et al., 2025; Yahiaoui et al., 2024; Yang et al., 2025; L. Zhang et al., 2025).

However, the diversity of FL architecture and analytic pipelines used across studies makes direct comparison difficult. Differences in model aggregation algorithms, encryption strategies, communication protocols, client-side configurations, and privacy-preserving add-ons contribute to inconsistent reporting and hinder the ability to standardize findings across the literature (Chen et al., 2024; J. Wang, 2025; Q. Wang et al., 2025).

### Theme 3: Encrypted Medical Imaging and Secure Training

The third theme captures research that applies encryption-based and cryptographic techniques to protect medical images during AI model development. The frequent appearance of terms such as *medical*, *imaging*, *encryption*, and *training* reflects the emphasis on securing data throughout the learning pipeline. Several studies demonstrate that models can be trained on encrypted inputs, using approaches such as homomorphic encryption and secure multi-party computation, while still achieving accuracy levels comparable to those trained on unencrypted data, although typically with increased computational demands (Son et al., 2021; Vizitiu et al., 2019). Work spanning cardiac monitoring, breast cancer diagnostics, and other imaging domains shows that strong encryption mechanisms can effectively protect patient information without severely compromising downstream model performance (Bai et al., 2025; Cao et al., 2023; L. Chen et al., 2024).

Another area of research focused on creating secure collaborative environments for AI development without sharing raw patient data. These efforts incorporate blockchain-enabled platforms, federated or distributed learning frameworks, and other privacy-preserving infrastructures designed to maintain data confidentiality while still supporting cross-institutional modeling efforts (Barnawi et al., 2024; Guan et al., 2023; Kaissis et al., 2021; Zerka et al., 2020). Collectively, these studies point to a growing interest in embedding cryptographic safeguards directly into medical AI workflows to reduce exposure risks and strengthen trust in distributed analytic systems.

### Theme 4: Healthcare Data Privacy and System Governance

The fourth theme captures the broader governance and organizational structures necessary to support privacy and security in healthcare AI systems. The recurring presence of terms such as *healthcare*, *system*, *security*, and *information* reflects the emphasis on institutional oversight rather than solely technical solutions. Across the included studies, researchers described governance approaches that integrate technical safeguards with organizational policies, for example, the use of privacy-impact assessments, audit trails, role-based access controls, encryption protocols, and secure logging to align AI operations with existing IT infrastructures (Danler et al., 2024; Sun et al., 2024).

A recurring challenge involves ensuring compliance with regulatory frameworks when AI systems span multiple institutions or areas. Studies noted that meeting the requirements of the Health Insurance Portability and Accountability Act (HIPAA), General Data Protection Regulation (GDPR), and region-specific privacy and security laws becomes increasingly complex as data moves across organizational boundaries (Dubey et al., 2025; Lee et al., 2024). Danler et al. (2024) specifically highlighted organizational strategies for bringing AI workflows into alignment with hospital IT policies, such as enforcing role-based permissions, implementing encryption standards, and maintaining tamper-resistant logs. Additional research also examined ethical and legal considerations associated with cross-site data flows, reinforcing that regulatory obligations are more difficult to satisfy when AI solutions are deployed in distributed environments (Dubey et al., 2025; Lee et al., 2024).

Beyond policy compliance, several studies explored how AI technologies operate within routine clinical information environments. These investigations discussed the integration of AI into electronic health records and health information exchange systems, alongside mechanisms such as federated learning, system-level monitoring, and well-defined data-stewardship responsibilities. Auditing tools and risk-management practices were also described as important mechanisms for preventing unintended disclosure and supporting accountable decision-making within AI-augmented processes (Om Kumar et al., 2023; Seyfizadeh et al., 2024). Collectively, this body of work underscores the increasing recognition that strong governance frameworks are essential complements to technical privacy controls, particularly in telehealth and telerehabilitation contexts where data often flows outside traditional institutional boundaries.

## Discussion

This systematic review brings together recent research addressing privacy and security concerns related to the use of AI in healthcare. The study literature is largely dominated by experimental studies, indicating a primary emphasis on the design and technical evaluation of AI-based solutions, with comparatively limited engagement in theory-driven or exploratory inquiry. Across studies, privacy protections receive more sustained attention than security-focused measures, including intrusion detection, threat surveillance, and resilience to adversarial attacks. Privacy and security are often discussed in tandem, which may conceal system-level weaknesses that are unique to AI-enabled clinical technologies. As a result, the body of evidence reflects a stronger orientation toward protecting sensitive information than toward safeguarding the operational integrity of AI systems deployed in clinical contexts.

These challenges are amplified in telehealth and telerehabilitation settings, where AI-supported services commonly function within decentralized, patient-managed environments rather than within centrally controlled institutional infrastructures. Remote monitoring technologies, wearable devices, and video-based care platforms generate continuous streams of sensitive data beyond traditional clinical boundaries. In home-based care scenarios, informed consent processes may become more difficult to implement effectively, particularly when patients are expected to understand complex privacy-preserving methods such as federated learning or differential privacy despite varying levels of technical familiarity.

A variety of technical strategies, including federated learning, differential privacy, blockchain-based architecture, encryption methods, and locally deployed language models, have been proposed to mitigate these risks. Although these approaches are well suited to decentralized and remote-care contexts, their application and performance within routine telehealth and telerehabilitation workflows remain inconsistently examined. Taken together, the findings point to persistent gaps in the practical integration of privacy and security mechanisms within remote-care ecosystems, offering important context for the detailed results, comparisons with prior work, and implications discussed in the sections that follow.

### Main Findings

Privacy and security concerns are particularly pronounced in telehealth and telerehabilitation settings, where continuous data capture, home-based monitoring technologies, and reliance on third-party platforms can increase exposure risks while reducing direct clinical oversight. Of the 80 studies included in this review, 66 focused on privacy-related issues, whereas only 17 explicitly examined security concerns, underscoring an imbalance in the current research landscape. Federated learning emerges as a frequently investigated strategy for enabling cross-institutional collaboration without the need to centralize patient data (Ain et al., 2023; Butt et al., 2023; Choudhury et al., 2025; Yan et al., 2023). Many studies further combine federated learning with techniques such as differential privacy and secure aggregation to limit the risk of sensitive information being inferred from model updates (He et al., 2024; Yang et al., 2025; L. Zhang et al., 2025).

Encryption remains a core strategy for securing health information in transit and at rest, while advanced methods, including homomorphic encryption and secure multi-party computation, support computation without direct exposure of raw data (P.-Y Chen et al., 2024; Kumar et al., 2023). Blockchain-based systems have also been explored as tools for enhancing data integrity, managing access permissions, and improving auditability within distributed clinical environments. Taken together, these findings show that most research continues to prioritize methods for protecting data confidentiality, whereas far fewer studies evaluate the operational security of AI systems once deployed in clinical settings.

### Comparison with Previous Research

Earlier scholarship consistently described privacy and security as foundational concerns in the use of AI within healthcare, frequently noting ethical challenges, regulatory uncertainty, public trust, and inconsistent adoption of available safeguards (Aggarwal et al., 2021; Chan et al., 2025; Chew & Achananuparp, 2022; Kelly et al., 2019; Khuller et al., 2022; Y. Li, et al., 2024; Tsoi, 2025). Across these studies, patient trust has been closely associated with transparent data practices and the presence of strong privacy protection. In contrast, the findings of the present review suggest a growing emphasis on system-level and architectural approaches that incorporate privacy and security directly into the design of AI-enabled healthcare technologies.

More recent work frames privacy engineering and governance as integral operational components, linking them to health-information exchange processes and model-performance considerations (Ragab et al., 2024; L. Zhang et al., 2025). Federated learning, once discussed primarily as a conceptual tool (Shojaei et al., 2024), is now more commonly implemented in multi-layered defense strategies aimed at protecting data while facilitating data analysis (Ain et al., 2023; Baumgartner et al., 2023; Butt et al., 2023; Hasan & Rahman, 2025; Li et al., 2025; Yahiaoui et al., 2024; Yan et al., 2023). Governance models have likewise evolved from abstract principles toward operational mechanisms, including access control systems, AI-enabled monitoring tools, and blockchain-supported electronic health records (Anita et al., 2025; Hennebelle et al., 2024; Ibrahim et al., 2024; Supraja et al., 2025). Taken together, this progression reflects an increasingly integrated approach in which privacy and security are addressed across the entire AI lifecycle to support scalable, trustworthy innovation in healthcare.

### Implications for Future Practice and Research

The findings of this systematic review indicate that privacy and security weaknesses persist in AI-enabled healthcare systems, even when data have been de-identified. This reality highlights the importance of embedding privacy-preserving strategies, such as federated learning, differential privacy, encryption, and secure computation, directly into clinical AI workflows. These protections are especially crucial in telehealth and remote-monitoring environments, where data are generated, transmitted, and processed outside the safeguards typically present in controlled clinical settings.

However, technical safeguards on their own are insufficient. Variability in data quality, institutional processes, and patient populations across decentralized systems can introduce bias, compromise model stability, and degrade diagnostic performance, particularly within federated learning architectures. Ensuring safe and scalable implementation will require healthcare organizations to establish coordinated governance frameworks that include standardized data-quality controls, clear procedures for model updating and aggregation, continuous performance auditing, and compliance with regulatory requirements such as HIPAA and GDPR.

Although encrypted training pipelines and secure imaging processes can further reduce exposure risk, wide-scale adoption is constrained by computational overhead and infrastructure limitations. Future work should prioritize real-world evaluations of these privacy-preserving methods, including assessments of their impact on model accuracy and reliability, as well as investigations into patient understanding of AI-related data protections to strengthen informed-consent practices.

### Study Limitations

This review has several limitations. First, the geographic representation was uneven, with most studies conducted in China, India, and Saudi Arabia, and few from the U.S, which may limit generalizability across different regulatory and cultural contexts. Second, many studies used experimental designs in non-healthcare settings, reducing insight into practical applicability. Third, the diversity of AI models (e.g., Convolutional Neural Network (CNN), Generative Adversarial Network (GAN), etc.) and privacy tools (e.g., differential privacy, blockchain) made direct comparisons problematic. Fourth, limited reporting on security measures may bias findings. Fifth, there was incomplete reporting of several study variables, which may introduce a reporting bias. Finally, limiting studies to English may introduce language bias and may exclude relevant research.

## Conclusions

This systematic review highlighted four key privacy and security themes in healthcare AI, showing a strong focus on privacy techniques like federated learning but limited attention to security measures and real-world use. Strengthening trustworthy AI will require multi-layered governance, consistent standards, broader geographic representation, and closer alignment between system design and health data practices.

## Figures and Tables

**Table 1:** Descriptive Characteristics of Included Studies (n=80)

Characteristics	Number	Percent (%)
**Study Design**		
Experimental	45	56.2
Model Development	12	15.0
Case Study	4	5.0
Pilot Study	4	5.0
Cohort	3	3.8
Cross-sectional	3	3.8
Algorithmic Development	2	2.5
Qualitative	2	2.5
Case-Control	1	1.2
Exploratory	1	1.2
Infrastructure Evaluation	1	1.2
Mixed Methods	1	1.2
Not Available	1	1.2
**Study Site**		
Non-healthcare Setting	37	46.3
Other	16	20.0
Hospital	14	17.5

Not Available	13	16.3

*Note.* “Not Available” reflects incomplete reporting by the authors.

**Table 2:** Geographic Distribution by Frequency

Country	Number
China	25
India	16
Saudi Arabia	10
United States	6
Canada	5
Germany	5
South Korea	4
Bangladesh	2
Finland	2
Jordan	2
Malaysia	2
Netherlands	2
Pakistan	2
Taiwan	2
Algeria	1
Australia	1
Austria	1
Belgium	1
Hong Kong	1
Oman	1
Portugal	1
Romania	1
Slovakia	1
South Africa	1
United Arab Emirates	2
Turkey	1
United Kingdom	1
Yemen	1

## Appendix

**Table A1:** Summary Table of Research Journals

(Authors, Year) *Journal*	Privacy	Security	Country/Region	Study Design	Study Site
(Aanjankumar et al., 2025) *IEEE Access*	Privacy-preserving healthcare data processing framework using differential privacy		India	Experimental	Hospital
(Abdalla et al., 2020) *Journal of Medical Internet Research*	Evaluated privacy risks of word embeddings trained on deidentified clinical notes, and found that sensitive patient information can be reconstructed from embeddings even after PHI removal.		Canada	Experimental	Clinic; Hospital
(Ain et al., 2023) *Diagnostics*	Proposed a federated learning–based skin cancer prediction framework that improves classification accuracy while preserving patient data privacy by avoiding centralized data collection.		Saudi Arabia	Case Study	Other
(Akmese, 2024) *BMC Medical Informatics and Decision Making*		Utilized encrypted biomarkers to ensure confidentiality to predict individuals with pancreatic ductal adenocarcinoma	Turkey	Case-Control	Hospital
(Alabdulwahab et al., 2024) *Sensors*	Synthetic data generation methods using a conditional tabular generative adversarial network (CT-GAN) aimed at maintaining the utility of IoT sensor network data for intrusion-detection systems (IDS) while safeguarding privacy.		Korea	Experimental	Non-healthcare Setting
(Almaiah et al., 2023) *Electronics*		Used a combination of IoTs data (EHRs, smart phones, smart watches, etc.) and an Encrypted Dynamic Bayesian Network) technique to secure the communication protocols so that only authorized people could access the data	Jordan, India & Saudi Arabia	Pilot Study	Other
(Almufareh, 2024) *IEEE Access*	Ensures privacy by performing real-time analysis and classification of skin lesion images directly on edge devices, minimizing data transmission and safeguarding sensitive patient information.		Saudi Arabia	Experimental	Clinic; Hospital
(Amoon et al., 2020) *Measurement*	Addressing privacy and security risks in healthcare data transmission and storage.	Applying AI-based spiral swarm optimized artificial immune system (SOAIS) to detect threats and ensure data integrity. Evaluating performance against malware and unauthorized access.	Saudi Arabia	Experimental	Other
(Anita et al., 2025) *Computer Methods and Programs in Biomedicine*		Developed multimodal user authentication.	India	Model Development	Non-healthcare Setting
(Arafat et al., 2025) *IET Blockchain*	Presents a blockchain-based framework for secure, scalable, and efficient sharing of medical images, prioritizing data integrity and privacy.	Presents a blockchain-based framework for secure and efficient sharing of medical images, prioritizing data integrity and privacy.	Bangladesh & Finland	Experimental	Non-healthcare Setting
(Arasteh et al., 2024) *Radiology: Artificial Intelligence*		Studied privacy-safe medical AI performance for X-rays	Germany	Experimental	Clinic; Hospital
(Bai et al., 2025) *Computer Methods and Programs in Biomedicine*		Propose PPCNN, a practical and privacy-preserving framework for CNN Inference, to enable secure sharing of body-related radiological images for deep learning.	China	Model Development	Non-healthcare Setting
(Bangare et al., 2024) *Frontiers in Health Informatics*	Evaluated federated transfer learning models under IID and non-IID conditions, highlighting federated learning’s effectiveness for distributed medical data without centralizing sensitive information.		India	Experimental	Non-healthcare Setting
(Barnawi et al., 2024) *IEEE Transactions on Network and Service Management*	Proposed a framework that combines Federated Learning (FL) and Differential Privacy (DP) to preserve the privacy of chest X-ray datasets used to develop and analyze high-performing Convolutional Neural Networks (CNNs) for detecting Tuberculosis.		Saudi Arabia	Experimental	Non-healthcare Setting
(Baumgartner et al., 2023) *Journal of Healthcare Informatics Research*	Compared decentralized learning methods for ECG classification and demonstrated that federated learning provides strong privacy protection with minimal performance loss.		Austria	Pilot Study	Non-healthcare Setting
(Bavli et al., 2025) *AI & Society*	Ethical implications of AI health monitoring for Parkinson’s disease. Stakeholder views on data privacy, ownership, and access to health data, use and potential misuse of data, and personal privacy at home.	Ethical implications of AI health monitoring for Parkinson’s disease. Stakeholder views on security, ownership, and access to health data, use and potential misuse of data, and personal privacy at home.	Canada	Qualitative	Other
(Benouis et al., 2024) *JMIR Mental Health*	To address privacy concerns with the sensitive data collected by wearable sensors, a framework that integrates differential privacy and federated learning approaches with Multi-Task Learning (MTL was used.		Germany	Experimental	Non-healthcare Setting
(Bergen et al., 2024) *Computer Methods and Programs in Biomedicine*	Proposed a 3-D generative model, Transversal GAN (TrGAN), to generate medical images that retain statistical properties and image features of the training data while balancing privacy and utility.		Canada	Experimental	Other
(Bharath et al., 2024) *The Open Public Health Journal*		Employed wavelet transform techniques to compress the Region of Interest (ROI) within medical images to retain the integrity of diagnostic information while reducing file size, improving performance for telemedicine.	India	Experimental	Clinic; Hospital
(Bi et al., 2022) *IEEE Transactions on Industrial Informatics*		Separate private data, analyze health information securely in a cloud system, and construct a security module based on the convolutional neural network.	China	Pilot Study	Other
(Butt et al., 2023) *Electronics*	Proposed a collaborative federated learning architecture for COVID-19 screening via chest X-ray images that preserves patient privacy by keeping imaging data localized across institutions.		Saudi Arabia	Model Development	Non-healthcare Setting
(Om Kumar C.U. et al., 2023) *Knowledge-Based Systems*		Designed a federated learning (FL) framework for secure EHR sharing across hospitals	India	Experimental	Other
(Cao et al., 2023) *Computers & Security*	Proposed a privacy-preserving healthcare monitoring framework and interactive protocols based on long short-term memory (LSTM) for edge servers.		China	Experimental	Non-healthcare Setting
(Chen, Cheng, et al., 2024) *IEEE Access*		Proposed a symmetric encryption and decryption protocol to ensure the security of biosignals and medical images and assist in disease diagnosis using a small-scale cascaded CNN architecture.	Taiwan	Experimental	Hospital
(Chen, Song, et al., 2024) *Image and Vision Computing*	Introduced Privacy-SF, an encoding-based framework for privacy-preserving medical image segmentation that protects sensitive features during both training and inference.		China	Experimental	Hospital
(Choudhury et al., 2025) *JMIR AI*	Developed and demonstrated a federated deep learning infrastructure—the Personal Health Train (PHT)—for training deep learning models on medical imaging data without sharing patient-level data across institutions.		Netherlands	Infrastructure Evaluation	Clinic; Hospital
(Chuang et al., 2025) *Journal of the American Medical Informatics Association*	Evaluated privacy risks and data utility trade-offs in using LLMs—GPT-3.5, GPT-4, and Mistral 7B—to generate synthetic EHR notes.		United States & Canada	Experimental	Hospital
(Deepak et al., 2024) *Measurement: Sensors*	Federated deep learning (FL) framework detected stress from wearable heart activity signals with accuracy comparable to traditional centralized methods (FL: 81.75%, centralized: 81.65%) while preserving privacy.		India	Experimental	Non-healthcare Setting
(Dubey et al., 2022) *International Journal of Electronics and Telecommunications*		Sensitive data stays local; advanced privacy methods ensure strong compliance with regulations.	India	Experimental	Other
(Dubey et al., 2025) *Computer Methods in Biomechanics and Biomedical Engineering: Imaging and Visualization*	Used federated learning for real-time mental health diagnostics by integrating multimodal data (physiological signals, online activity, social media interactions).		India & South Africa	Experimental	Non-healthcare Setting
(Ellis et al., 2025) *Journal of the American Geriatrics Society*	Privacy concerns regarding information shared with novel patient-facing chatbots.		United States	Mixed Methods	Clinic; Hospital
(Ferdowski et al., 2024) *Computer Methods and Programs in Biomedicine*		Uses differential privacy and data anonymization to protect sensitive attributes, ensuring secure and ethical AI-based cardiovascular disease prediction.	United States, Hungary, & Switzerland	Experimental	Other
(Gong et al., 2025) *IEEE Journal of Biomedical and Health Informatics*	Experimental performance evaluation using real-world cases from medical question-answering datasets; comparison with existing large language models for accuracy.		China	Case Study	Non-healthcare Setting
(Guan et al., 2023) *International Journal of Surgery*	Presented machine learning (ML) models to predict the lymph node metastasis (LNM) status for rectal cancer patients before surgery based on a privacy-preserving computing platform (PPCP).		China & United States	Cohort	Hospital
(Gulati et al., 2025) *International Journal of Mathematical, Engineering and Management Services*	Applied federated learning to ocular disease detection, demonstrating accurate diagnosis while maintaining patient privacy by avoiding centralized data storage.		China	Experimental	Non-healthcare Setting
(Guo et al., 2020) *Computers & Security*	Proposed a classification and retrieval method to search for and locate relevant cases by constructing a privacy-preserving Convolutional Neural Network (CNN) framework that allows the classification and searching of secure, content-based, large-scale encrypted images with homomorphic encryption.		China	Experimental	Non-healthcare Setting
(Haque et al., 2023) *Journal of Network and Computer Applications*	Security model development and testing.		China	Model Development	Non-healthcare Setting
(Haripriya et al., 2025) *Scientific Reports*	Integrated transfer learning with federated learning to enable scalable and privacy-preserving medical image classification across multiple institutions.		India	Model Development	Hospital; Other
(Hasan et al., 2025) *Smart Health*	Designed a differentially private federated learning system for polyp segmentation that achieves high accuracy without exposing sensitive patient data.		Bangladesh	Model Development	Hospital
(He et al., 2024) *Future Generation Computer Systems*	Proposed a differentially private swarm learning framework using generative augmentation to address non-IID clinical data while providing formal privacy guarantees.		China	Experimental	Clinic; Hospital
(Hennebelle et al., 2024) *Computational and Structural Biotechnology Journal*		Developed a secure, privacy-preserving, end-to-end system integrating IoT, edge computing, AI, and blockchain for predicting type 2 diabetes based on risk factors.	Australia, United Arab Emirates, & India	Experimental	Non-healthcare Setting
(Hossen et al., 2023) *IEEE Journal of Biomedical and Health Informatics*	Evaluated a federated learning–based CNN framework for skin disease classification that ensures patient data privacy while improving model generalization across distributed clients.		New Zealand	Pilot Study	Clinic; Hospital
(Huang et al., 2023) *Biomedical Signal Processing and Control*	Proposed a deep learning–based zero-watermarking scheme for medical images that enhances protection against tampering and attacks through encryption and robust watermark security.		China	Experimental	Clinic; Hospital
(Ibrahim et al., 2024) *Sensors*	Implementation and evaluation of an ensemble intrusion detection system using honeypot diversion and machine learning.		Jordan	Experimental	Non-healthcare Setting
(Inam et al., 2024) *Egyptian Informatics Journal*	Proposed Cycle_GAN (Cycle-Consistent Generative Adversarial Network) to protect the confidentiality of unpaired medical images used in deep learning.		Pakistan & Saudi Arabia	Experimental	Non-healthcare Setting
(Iqbal et al., 2025) *Alexandria Engineering Journal*	Introduced a domain-adaptive federated learning framework combined with edge computing to enable privacy-preserving multi-modal medical image analysis across heterogeneous institutions.		China, Pakistan, Korea, Canada, India, & Saudi Arabia	Experimental	Non-healthcare Setting
(John & Kumar, 2023a) *Measurement: Sensors*	Proposed an encryption scheme using a linear feedback shift register (LFSR) for DICOM CT images to be securely and efficiently shared through the cloud for telemedicine applications.		Malaysia & India	Algorithmic Development	Non-healthcare Setting
(John et al., 2023b) *Procedia Computer Science*	Proposes a hybrid chaotic model combining the 2D Lorentz Chaotic model with the Logistic chaotic model for secure encryption/decryption of DICOM CT images through the cloud to improve teleradiology applications.		Malaysia	Experimental	Other
(Kaissis et al., 2021) *Nature Machine Intelligence*	Presented PriMIA (Privacy-preserving Medical Image Analysis), a framework for differentially private, securely aggregated federated learning and encrypted inference on medical imaging data.		Germany	Case Study	Other
(Kokin et al., 2024) *Procedia Computer Science*	Investigated how differential privacy can optimize both privacy and model performance during deep-learning training, specifically in sensitive medical data applications (heart disease).		Slovakia	Model Development	Non-healthcare Setting
(Kolhar & Misfer, 2023) *Intelligent Automation & Soft Computer*	Proposes a process to combine machine learning and cloud computing to protect patient and provider data when sharing heart rate data streams to predict myocardial infarction.		Saudi Arabia	Experimental	Non-healthcare Setting
(Lee et al., 2024) *Information Sciences*	Federated learning framework (HarmoSATE) that improved predictive accuracy using harmonized contextual embeddings and self-attention mechanisms across EHR data		Republic of Korea	Experimental	Hospital
(Li, Peng, et al., 2024) *Telemedicine and e-Health*	Investigates predictors of adoption and continued use of AI chatbots for mental health among U.S. adults with depression and anxiety symptoms.		United States	Cross-sectional	Other
(Li et al., 2025) *Biomedical Signal Processing and Control*	Demonstrated that federated learning enables accurate sleep stage classification while protecting patient data and mitigating class imbalance across distributed clients.		China & Finland	Experimental	Clinic; Hospital
(Liu et al., 2022) *Computers in Human Behavior*	Investigates how trust and AI-related features affect behavioral intention to use smart healthcare services, and how these relationships vary by gender, age, and usage experience.		China	Cross-sectional	Other
(Montenegro et al., 2021) *IEEE Access*	Proposed a privacy-preserving generative adversarial network for the privatization of case-based explanations, guaranteeing privacy, intelligibility, and preservation of explanatory evidence using interpretability saliency maps to reconstruct relevant features.		Portugal	Model Development	Non-healthcare Setting
(Nowak et al., 2025) *Radiology*	Publicly available and privacy ensuring LLMs can perform just as well as expensive proprietary systems like GPT-4o for organizing information from doctors’ radiology reports.		Germany	Exploratory	Hospital
(Ragab et al., 2024) *Human-centric Computing and Information Sciences*	Development of a blockchain privacy preserving technique for EHR analysis and for storing of patients’ clinical and institutional data.		Saudi Arabia	Model Development	Non-healthcare Setting
(Sangaiah et al., 2024) *IEEE Transactions on Engineering Management*	Enhanced privacy protection in healthcare data mining by applying k-anonymity and entropy-based strategies to anonymize sensitive patient information in distributed IoT environments.		Taiwan	Algorithmic Development	Non-healthcare Setting
(Seyfizadeh et al., 2024) *Expert Systems with Applications*		Used a deep residual neural network (ResNet) for the identification of individuals based on their EEG signals, to enhance security in brain-computer interface (BCI) applications through improved user identification accuracy and superior performance over other methods.	Germany	Experimental	Non-healthcare Setting
(Shao et al., 2020) *JMIR Formative Research*	Developed and validated a privacy-preserving federated learning method that enables distributed model training without sharing sensitive medical data.		China & U.S.	Experimental	Other
(Son et al., 2021) *PLoS ONE*	Proposed a method for privacy-preserving Gated Recurrent Unit (GRU) inference model using privacy-enhancing technologies, including homomorphic encryption and secure two-party computation in the prediction of breast cancer.		South Korea	Cohort	Other
(Supraja et al., 2025) *Biomedical Engineering Applications Basis and Communications*	.	Secure transmission of patient data via IoT sensors	India	Experimental	Non-healthcare Setting
(Tang et al., 2023) *Biomedical Signal Processing and Control*	Proposed a compressed sensing–based transformer network for medical image segmentation that reduces data exposure while maintaining performance under strict data protection regulations.		China	Model Development	Non-healthcare Setting
(Tang et al., 2024) *IEEE Journal of Biomedical and Health Informatics*	Developed a privacy-preserving federated learning framework with domain adaptation for ocular disease recognition using Gaussian noise mechanisms to protect local data.		Hong Kong & China	Model Development	Non-healthcare Setting
(Teo et al., 2025) *Ophthalmology*	Proposed federated learning algorithm applying blockchain to overcome privacy shortcomings when transferring images for the detection of myopic macular degeneration.		China	Cohort	Non-healthcare Setting
(Vignesh et al., 2025) *Procedia Computer Science*	AI models (including BERT, a language processing system) combined with autoencoders to achieve 93% accuracy in identifying mental health conditions while keeping PHI protected.		India	Experimental	Other
(Vizitiu et al., 2019) *2019 41st Annual International Conference of the IEEE Engineering in Medicine and Biology Society (EMBC)*	Proposed a solution, based on the MORE homomorphic encryption scheme, to allow deep learning models to maintain confidentiality of medical image data while enabling computations.		Romania	Model Development	Non-healthcare Setting
(Wang, S. et al., 2025) *Frontiers in Medicine*	Developed a differential privacy–based deep reinforcement learning (DP-DRL) framework for sepsis treatment, particularly in patients with pulmonary complications		China	Experimental	Non-healthcare Setting
(Wang, Q., 2025) *Engineering Applications of Artificial Intelligence*	Proposed an interpretable vertical federated learning framework that integrates private multi-source data to improve cancer prognosis prediction accuracy without sharing raw data.		China	Experimental	Non-healthcare Setting
(Wang, J., et al., 2025) *Information Fusion*	Introduced PHMS-Fed, a privacy-preserving federated learning framework for heterogeneous multi-modal sensor fusion in smart healthcare systems.		China	Experimental	Non-healthcare Setting
(Xu et al., 2022) *IEEE Transactions on Industrial Informatics*	Developed a federated learning framework for detecting depression using multisource mobile health data while preserving patient privacy.		China	Experimental	Non-healthcare Setting
(Yahiaoui et al., 2024) *Diagnostics*	Presented a privacy-aware federated learning framework with differential privacy techniques for distributed brain tumor segmentation without compromising data confidentiality.		Algeria, Oman, United Arab Emirates, & Yemen	Experimental	Non-healthcare Setting
(Yan et al., 2023) *Electronics*	Proposed a lightweight privacy-embedded federated learning model for breast tumor detection in IoMT environments that improves diagnostic accuracy while protecting patient data.		China	Experimental	Non-healthcare Setting
(Yang et al., 2025) *Biomedical Signal Processing and Control*	Developed a privacy-preserving federated domain generalization framework for multi-center eye imaging using synthetic data generation and secure global aggregation to improve model robustness across domains.		China	Experimental	Non-healthcare Setting
(Zarifis et al., 2021) *Journal of Internet Commerce*	Investigates how visible AI involvement in health insurance purchasing affects: trust in the system and privacy concerns about sharing sensitive health and financial data.		United Kingdom	Cross-sectional	Other
(Zerka et al., 2020) *IEEE Access*	Demonstrated effectiveness of Chained Distributed Machine learning (C-DistriM), combining sequential distributed learning with blockchain to provide transparency and increase trust in AI computations on medical images.		Netherlands & Belgium	Case Study	Hospital
(Zhang et al., 2022) *Information Fusion*	Proposes an image release scheme, QAPP, which integrates the discrete cosine transform (DCT) with differential privacy (DP) to maintain confidentiality of high-quality medical images.		China	Experimental	Hospital
(Zhang et al., 2025) *JAMIA Open*	Developed and evaluated LLMs (Axpert) for automated labeling of pediatric abdominal X-ray (AXR) reports, for necrotizing enterocolitis (NEC) diagnosis, and subtype classification shows that Axpert outperforms baseline models.		United States	Qualitative	Hospital
(Zhang et al., 2025) *Cluster Computing*	Introduced FedCCW, a Byzantine-robust and privacy-preserving federated learning scheme using differential privacy and secure aggregation to enable safe multi-institutional medical collaboration.		China	Experimental	Non-healthcare Setting
